# Early burn wound excision in mass casualty events

**DOI:** 10.1186/s40779-022-00407-x

**Published:** 2022-08-09

**Authors:** Agnieszka Surowiecka, Tomasz Korzeniowski, Jerzy Strużyna

**Affiliations:** 1 East Center of Burns Treatment and Reconstructive Surgery, 21-010 Łęczna, Poland; 2 Chair and Department of Didactics and Medical Simulation, Medical University of Lublin, 20-093 Lublin, Poland; 3 Department of Plastic Surgery, Reconstructive Surgery and Burn Treatment, Medical University of Lublin, 20-093 Lublin, Poland

**Keywords:** Burn wound, Early excision, Enzymatic debridement

Dear Editor,

The aim of the letter is to stress the need of creating unified recommendations concerning early burn wound excision in cases of mass casualty burns exclusively. There are several triage methods, both for civilian and military circumstances, that govern burn casualty triage and evacuation from the incident site, as well as hospital referral up to 120 h from the disaster. Most burn response plans allow for burn surgery to be delayed even for several days until secondary triage is performed and the patient is transferred to a burn centre. However, data regarding further management of burn casualties is limited.

The act of the first responders is limited to evacuation and implementation of proper fluid resuscitation, but there are various unpredictable factors, like geographical location, number of rescuers available, and type of the event, that might influence the operation. In austere circumstances and in mass casualty events the number of burned patients may exceed the abilities of the first responders. This may lead to delay of first aid as well as delay in definitive surgical treatment. World Health Organization (WHO) recommendations for early wound care include performing life-saving procedures such as escharotomy, fasciotomy, and wound cleaning at the first receiving hospital. We need to stress that, most burn patients are treated at non-burn hospitals, with limitation of burn specific equipment and trained burn surgeon and nurses. The absence of unified recommendations regarding surgical treatment of the burn wound is a serious problem. Early burn wound excision performed within the first 48 h postburn significantly reduces mortality and in-hospital stay even in the case of major burns. Early surgical intervention and eschar removal decrease mortality by reducing bleeding, infection risk, and energy demand. Delay in necrectomy and skin grafting leads to wound infection, and may result in serious septic complications.

Not only timing of surgery is important. Extent of excision, blood loss and availability of autologous or allogenic skin grafts are also crucial factors determining every necrectomy. Tangential and late excisions are characterized by higher blood loss in comparison to fascial excisions. Fascial technique involves resection of full-thickness burns with underlying skin and subcutaneous fat to the layers of fascia and well-vascularized tissues. In most cases, the extent of a single eschar removal procedure is up to 40% of total body surface, and usually within 20%. The ideal setting for burn wound management is a separate from abdominal surgery operating room, but mass casualty burn events may force medical teams to use other spaces in the ward or intensive care unit.

When definitive surgery is not available, wound control, pain management, and splinting preventing contractures are recommended. There were several reports from low- and middle-income countries stating that early aggressive surgical treatment might not be beneficial in extensive burns, and the risk of iatrogenic complications is high. This might be due to shortages of resources, including specialized equipment, antimicrobial dressings, as well as lack of trained and experienced personnel [1]. Non-surgical burn debridement can be performed with a proteolytic pineapple-derived enzyme, bromelaine [2]. The benefits of the ointment are that it removes necrolytic tissues and can be applied by a trained nurse or doctor, without the need for an operating room. Specific debridement with preservation of the reticular layer of the dermis significantly reduces the area and promotes self-healing [3]. In many cases, skin grafting and further surgical excisions are not necessary, and blood loss is less extensive than with traditional necrectomy. Pain control can be performed with intravenous analgesia [4]. The procedure requires prior moist preparation of the burn wound. Even though bromelaine is recommended for a single removal of 15% of the total body surface area, we have a successful experience of multistage, 2–6 times of 10–15%, enzymatic debridement of 60% total body surface area (TBSA). We have previously remarked that accurate evaluation of burn depth is feasible after enzymatic debridement, and the extent of burn wound requiring definitive closure might be reduced [5]. Non-surgical debridement might be an alternative to comprehensive treatment that relies on eschar separation. Application of an enzymatic ointment is time consuming, it requires less equipment, less blood supply, and the process of training is easier.

There is a strong need for personnel training as a part of burn disaster management plans. A trained burn surgeon in some institutions is recognized as, with at least 3 years or 5 years of experience in a burn unit. Some organisations allow telemedicine in a mean of supporting non-burn hospitals. Response plans for mass burn casualty events should include clear guidelines for early burn excision or debridement.

We hope our opinion will encourage decision makers to prepare detailed burn response plans that would include type and timing of burn wound excision.

## Data Availability

Not applicable.

## Abbreviations

WHO: World Health Organization
TBSA: Total body surface area
